# Timing of Organ Procurement From Brain-Dead Donors Associates With Short- and Long-Term Outcomes After Liver Transplantation

**DOI:** 10.3389/ti.2022.10364

**Published:** 2022-08-31

**Authors:** Verner Eerola, Ilkka Helanterä, Fredrik Åberg, Marko Lempinen, Heikki Mäkisalo, Arno Nordin, Helena Isoniemi, Ville Sallinen

**Affiliations:** Department of Transplantation and Liver Surgery, Helsinki University Hospital and the University of Helsinki, Helsinki, Finland

**Keywords:** graft survival, brain death, organ allocation, donor hepatectomy, liver allograft function, liver transplant dysfunction, procurement surgery

## Abstract

Brain death-induced cytokine storm is thought to harm transplantable organs. However, longer procurement times have been associated with non-inferior or better outcomes in kidney, heart, and lung transplants, while optimal procurement time for liver allografts is unknown. Our aim was to analyze the association of time interval from brain death to organ procurement with liver allograft outcomes in two nationwide cohorts. The association of procurement interval with graft survival and short-term complications was analysed in multivariable models. Altogether 643 and 58,017 orthotopic liver transplantations from brain-dead donors were included from Finland between June 2004 and December 2017 and the US between January 2008 and August 2018, respectively. Median time from brain death to organ procurement was 10.5 h in Finland and 34.6 h in the US. Longer interval associated with better graft survival (non-linearly, *p* = 0.016) and less acute rejections (OR 0.935 95% CI 0.894–0.978) in the US cohort, and better early allograft function (*p* = 0.005; Beta −0.048 95% CI −0.085 −(−0.011)) in the Finnish cohort, in multivariable models adjusted with Donor Risk Index, recipient age, Model for End-Stage Liver Disease and indication for transplantation. Progressive liver injury after brain death is unlikely. Rushing to recover seems unnecessary; rest and repair might prove beneficial.

## GRAPHICAL ABSTRACT

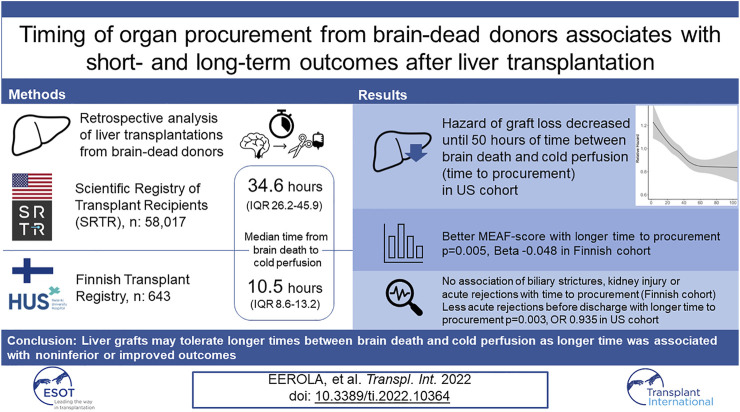

## Introduction

Majority (86%–100%) of liver transplants are still obtained from brain dead donors (1). The so called “cytokine storm” that follows brain death causes hemodynamic and blood coagulation changes leading to well described cell damage and ischaemia in various organs (2). Animal studies suggest organs from brain dead donors are harmed during and after brain death (2), and longer procurement interval (i.e., time interval from brain death to procurement) has led to increased inflammation, immune activation, and organ dysfunction (3–5). However, brain death is a continuous process and donor stabilization—“storm settling”—is usually achieved in a manner of hours (6) as care for the donor has been perfected over the decades up to nearly a routine. Effects of brain death and recovery of damage to the organs related to time of brain death are not well understood, and some transplant centers aim to procure as fast as possible. However, procurement intervals in US centers have grown gradually longer, without apparent harm in retrospective studies of transplanted kidneys, hearts and lungs (7–10). Of note, effect of procurement interval on liver allografts has not been studied.

Consequences of brain death may differ between organs and so might the optimal time-point of procurement, which for lungs and heart seems as long as possible, but for the kidneys between 20 and 50 h (9–11). Identifying the optimal time for procurement of liver grafts has implications both in transplantation logistics and outcomes. The simultaneous nature of abdominal organ procurement demands this effect to be studied in all organs.

This study aimed to examine the association of procurement interval with early liver allograft function and graft survival in two different transplant populations with different median times from brain death to organ procurement (Finland and the US). Associations with other important endpoints, such as acute rejections, biliary strictures, and post-operative kidney injury, available for the Finnish cohort, were also studied.

## Materials and Methods

### Donors and Patients

#### Finnish Cohort

All orthotopic liver transplantations from deceased donors performed in Finland between June 2004 and December 2017 were included and followed until death, retransplantation, or October 2020. The data were extracted from the Finnish Transplant Registry and donor medical records. Organs exchanged internationally were excluded from the study. All included organs were procured within Finland by the same team of transplant surgeons from Helsinki Transplantation and Liver Surgery Unit, and all transplantations in Finland were performed at Helsinki University Hospital. All liver grafts were donations after brain death (DBD) in Finland during the study period.

#### US Cohort

This study used data from the Scientific Registry of Transplant Recipients (SRTR). The SRTR data system includes data on all donors, wait-listed candidates, and transplant recipients in the US, submitted by the members of the Organ Procurement and Transplantation Network (OPTN). The Health Resources and Services Administration (HRSA), U.S. Department of Health and Human Services provides oversight to the activities of the OPTN and SRTR contractors. Orthotopic liver transplantations recorded in SRTR database in the US between January 2008 to August 2018 were included. Follow-up consisted of the same time-period. Only livers transplanted from DBD donors were included, and livers from donation after circulatory death (DCD) or living donors were excluded.

The clinical and research activities being reported are consistent with the Principles of the Declaration of Istanbul as outlined in the “Declaration of Istanbul on Organ Trafficking and Transplant Tourism” and the Declaration of Helsinki.

#### Variables

The following donor variables were collected for both Finnish and US cohorts: donor age and gender, the time of declaration of brain death, the start time of cold perfusion in organ procurement surgery, cause of death, body mass index, donor history of hypertension, diabetes and hepatitis C status. Regarding the recipient and transplantation, recipient age and gender, cause of end-stage liver disease, Model for End-Stage Liver Disease (MELD) score at listing and before transplantation, body mass index, history of hypertension, human leukocyte antigen mismatches, graft cold ischemia time, anhepatic time, use of partial graft, organ location, acute rejection episodes, and graft survival were collected. For the Finnish cohort usage of Molecular Adsorbent Recirculating System (MARS), hemodialysis prior to transplantation and additional follow-up data of post-operative dialysis, post-operative laboratory results, and biliary complications were collected. Donor Risk Index (DRI) was calculated from donor variables according to formula by Feng et al. (12) for both Finnish and US donors. Variables used to calculate DRI are donor age, cause of death, race, graft splitting, donor height, organ location, and cold ischemia time. All organs in Finland were defined as local. Race was not available for the Finnish cohort due to Finnish legislation, but as overwhelming majority of the Finnish population is Caucasian, all Finnish donors were considered Caucasian. Because the models included DRI, donor factors used to calculate DRI were left out from the multivariable models due to possible multi-collinearity. Procurement interval was defined as the time from the declaration of brain death to the start of *in situ* cold perfusion.

#### Endpoints

Model of Early Allograft Function (MEAF)-score was selected as the primary short-term outcome measure (13). Based on alanine aminotransferase, international normalized ratio, and bilirubin, MEAF-score defines liver function numerically from 0 to 10, 3 days after transplantation. Acute liver failures, transplantations for under 18-year-olds and split transplantations were excluded, because MEAF is validated only for full liver grafts, adults and for non-acute liver failures. Beta in the results is given by one MEAF point per 1 hour change in procurement interval. Missing International Normalized Ratio values for 42 cases were calculated from prothrombin time with a conversion table supplied by the laboratory (HUSLAB) responsible for the blood tests. Post-operative kidney injury was assessed with post-operative need of dialysis and also by any grade of kidney injury defined by the Kidney Disease Improving Global Outcomes (KDIGO) -guidelines within the first 7 days (14).

For the Finnish cohort, acute rejections were defined as the need for rejection treatment in a biopsy-proven borderline, or acute cellular, or antibody-mediated rejection. The risk of intrahepatic biliary strictures was also assessed since this complication is strongly associated with ischemia-reperfusion injury (15). Strictures were diagnosed with either endoscopic retrograde cholangiography (ERC) or with magnetic imaging where ERC was not possible or not done.

Acute rejections in the US cohort were recorded to the SRTR database by accuracy of whether patient had an acute rejection before discharge or before a follow-up date. Consequently, early acute rejections were defined as a rejection before discharge time. Acute rejections during first year were analyzed by patient having an acute rejection episode before discharge or before 1-year follow-up after transplantation. In the Finnish cohort, 30 days was considered the cut-off for early acute rejection.

Graft survival, in which graft failure was defined as a composite outcome of retransplantation or recipient death, was chosen as the long-term dependent outcome measure.

### Statistical Analysis

Transplantations were divided into tertiles based on procurement interval for graphical purposes. Characteristics of data and groups are reported with median and interquartile range (IQR) for continuous data and frequencies with percentages for categorical data in the tables. Number of patients with missing values are stated in Table 1.

**TABLE 1:** Characteristics and outcomes of liver transplantations in Finland from June 2004 to December 2017 and the US from January 2008 to August 2018.

Variable	Finland N: 643	US N: 58 017	Missing FIN	Missing US
Donor				
Procurement interval, hours	10.5 (8.6–13.2)	34.6 (26.2–45.9)	0	0
Donor age, years	53 (41–61)	38 (24–52)	0	0
Donor BMI, kg/m^2^	24.5 (22.7–26.9)	26.1 (22.6–30.4)	0	0
Donor gender, male	342 (53.2%)	34,591 (59.6%)	0	0
Donor medical history				
Hypertension	170 (26.4%)	18,403 (31.7%)	0	339 (0.6%)
Diabetes	37 (5.8%)	5,729 (9.9%)	0	0
Donor cause of death:			0	0
Anoxia	16 (2.5%)	17,773 (30.6%)		
Cerebrovascular accident	443 (68.9%)	18,833 (32.5%)		
Trauma	163 (25.3%)	19,985 (34.4%)		
Other	21 (3.3%)	1,426 (2.5%)		
Donor Risk Index (DRI)^a^	1.46 (1.22–1.68)	1.27 (1.08–1.52)	7 (1.1%)	557 (1.0%)
Donor organ yield^b^	3 (3–4)	3 (3–4)	0	0
More than liver and kidney donor^c^	258 (40.1%)	31,664 (54.6%)	0	0
Thoracic organ donor	204 (31.7%)	29,806 (51.4)	0	0
Donor cardiac arrest prior to brain death	98 (15.2%)	3,868 (6.7%)	0	0
Donor race, caucasian	NA	44,764 (77.2%)	NA	0
Recipient				
Partial/split graft	55 (8.6%)	1,465 (2.5%)	0	0
Cold ischemia, hours	4.9 (4.3–5.7)	6.1 (4.8–7.8)	7 (1.1%)	557 (1.0%)
Recipient age at transplantation, years	52 (37–60)	56 (47–62)	0	0
Recipient gender, male	350 (54.4%)	37,885 (65.3%)	0	0
Retransplantation	57 (8.9%)	3,637 (6.3%)	0	0
Combination transplantation, kidney	28 (4.4%)	5,917 (10.2%)	0	0
Median waiting time, days	24 (6–61)	83 (15–274)	0	0
Usage of MARS	50 (9.3%)	NA	105 (16.3%)	NA
Anhepatic time, minutes	57 (51–65)	NA	6 (0.9%)	NA
Total bleeding, litres	2.5 (1.5–4.5)	NA	7 (1.1%)	NA
MELD at transplantation	15.2 (10.5–21.4)	21 (13–31)	82 (12.8%)	0
Indication for transplantation			0	10 (0.0%)
Acute liver disease	80 (12.4%)	3,002 (5.2%)		
Chronic liver disease	463 (72.0%)	45,924 (79.2%)		
Metabolic liver disease^d^	22 (3.4%)	1,767 (3.0%)		
Tumor	78 (12.1%)	7,314 (12.6%)		
Primary liver pathology			0	0
Acute liver failure	80 (12.4%)	2,633 (4.5%)		
Primary sclerosing cholangitis	108 (16.8%)	2,496 (4.3%)		
Primary biliary cirrhosis	50 (7.8%)	1,335 (2.3%)		
Malignancy^e^	101 (15.7%)	10,392 (17.9%)		
Alcoholic liver disease	104 (16.2%)	10,730 (18.5%)		
HCV cirrhosis	16 (2.5%)	11,983 (20.7%)		
NASH	19 (3.0%)	5,857 (10.1%)		
Other	168 (26.1%)	12,591 (21.7%)		
Graft survival^f^			0	0
1-year	91.6%	88.1%		
2-year	87.8%	83.7%		
3-year	85.2%	80.3%		
5-year	80.8%	74.4%		
10-year	71.5%	59.5%		
15-year	55.1%	NA		
Model of Early Allograft Function-score	3.2 (1.9–4.4)	NA	7 (1.4%)	NA
Intrahepatic biliary stricture	31 (4.8%)	NA	2 (0.3%)	NA
Anastomotic biliary stricture	91 (14.2%)	NA	0	NA
Biliary leak	18 (2.8%)	NA	1 (0.2%)	NA
Early acute rejection	152 (23.6%)	3,102 (5.4%)	0	51 (0.1%)
Acute rejection during first year	231 (35.9%)	3,418 (14.6%)^g^	0	0^g^
Dialysis after transplantation^h^	146 (22.7%)	NA	0	NA
Post-operative kidney injury^i^	366 (68.0%)	NA	1 (0.2%)	NA
Grade 1	151 (28.1%)			
Grade 2	88 (16.4%)			
Grade 3	127 (23.6%)			
Follow-up time, years	6.6 (3.3–10.7)	2.9 (1.0–5.8)	0	0

a Formula by Feng et al. (12).

b Number of organs donated per donor.

c Donor donated organs besides liver and kidneys.

d Metabolic liver disease by definition of Scientific Registry of Transplant Recipients (e.g., Wilson’s disease, hemochromatosis, alpha-1 antritrypsin deficiency, thyrosinemia, primary oxalosis, hyperlipidemia; does not include nonalcoholic fatty liver disease).

e Malignancy in removed liver, indication in some cases has been other (e.g., PSC or alcoholic cirrhosis) prior to transplantation, overrules other primary diagnoses.

f Graft survival defined as combination outcome of death or retransplantation.

g Sub-cohort of 23,430 patients with sufficient data from 2013 to 2018.

h Includes all patients after transplantation.

i Acute kidney injury defined by KDIGO guidelines, 104 patients excluded from analysis because of preoperative dialysis.

All values are stated as median (interquartile range) or categorical data as exact number (percentage of all) unless otherwise indicated.

NA, data not available for US cohort.

Potential confounders to analysis were identified by a directed acyclic graph (DAG) (16). The DAG presentation (Supplementary Figure S1) explains our team’s understanding of factors affecting the analysis, which were considered the same for all endpoints. From the DAG we identified DRI, patient age, patient MELD and indication of acute liver failure as confounders. The association between procurement interval (hours) and MEAF were assessed with a linear regression model (ordinary least squares). Cox proportional hazards models were used to analyze association of procurement interval on graft survival. The association of procurement interval with post-operative kidney injury and kidney injury requiring dialysis was assessed with logistic regression models after excluding preoperatively dialyzed patients. Logistic regression was used to analyze the association of procurement interval with biliary strictures and with acute rejections. Potential confounders were controlled with complete-cases data in all analyses and cases with missing variables were excluded.

Restricted cubic spline functions were used to account for potentially non-linear association between the outcome of interest and procurement interval and confounders, as the linear regression, logistic regression and Cox models involve the assumption of linearity for continuous data. Non-linearity was tested for, and the associations were modelled either as linear or non-linear. Linear associations between procurement interval and the outcome of interest were reported using the beta, odds ratio (OR) or hazard ratio (HR) with 95% confidence interval (CI), as appropriate. Non-linear results are reported with *p*-values and figures for clarity. The associations analyzed with spline functions were reported by plotting the predicted relative hazard of graft survival or endpoint as a function of procurement interval. The proportional hazards assumption for procurement interval was checked using Schoenfeld residuals, and no violations were detected. Effort to limit bias was addressed by sparse exclusion criteria, testing all endpoints for non-linearity and adjusting for possible confounders. Sensitivity analyses by donor organ yield and year of transplantation were conducted to account for possible confounding.

The significance level was set at 5% and analyses were carried out as two-tailed. All analyses were performed using either IBM SPSS version 27 for Windows (Armonk, NY), or R software, including survival and rms packages (R Foundation for Statistical Computing, Vienna, Austria).

## Results

### Patients

Altogether 721 and 73,222 orthotopic liver transplantations were performed during the time periods in Finland and the US, respectively. In the Finnish cohort, 77 transplantations were excluded as the graft was received from another country and one was lost to follow-up resulting in 643 transplantations in the final Finnish cohort. From the US cohort, 3,104 living and 3,737 DCD donors were excluded from the analysis. In addition, extreme procurement interval values of over 120 h (203 donors) and under 2 hours (eight donors) were excluded for unreliability of brain death time. Also, transplantations with missing time of brain death, follow-up time or status (8,153 transplantations) were excluded, leaving 58,017 transplantations in the US cohort altogether.

**FIGURE 1:**
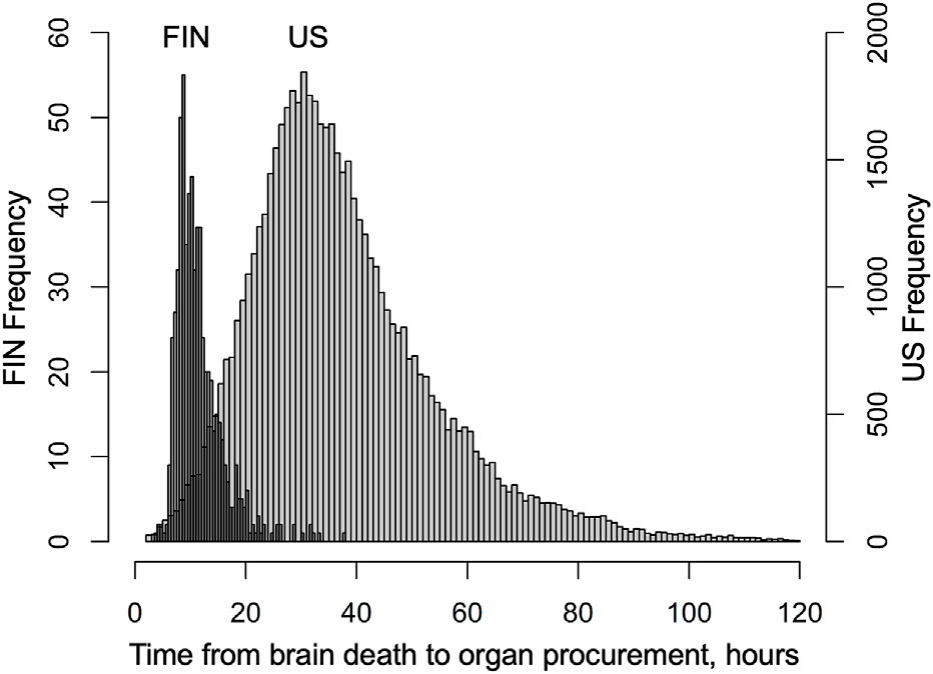
Distribution of time from declaration of brain death to organ procurement (procurement interval) in Finnish liver donors from June 2004 to December 2017 and SRTR liver donors from January 2008 to August 2018.

Median interval from brain death to cold perfusion was 10.5 h in Finland and 34.6 h in the US. Distribution of these procurement intervals are presented in Figure 1. During follow-up, 131 and 11,396 patients died, and 42 and 1,509 were retransplanted in Finland and the US, respectively. Characteristics of donors, transplantations and patients in both cohorts are summarised in Table 1, which also includes follow-up data of complications in the Finnish cohort and numbers of missing values. Characteristics are divided by procurement interval tertiles in Table 2 and outcomes in Table 3.

**TABLE 2:** Characteristics of liver transplantations in Finland and the US by tertiles of time between brain death and organ procurement (interval).

Variable	Finland 6/2004–12/2017			US 1/2008–8/2018		
Donor						
Tertile of procurement interval	1st < 9.2 h n:214	2nd 9.2–12.0 h n:215	3rd > 12.0 h n:214	1st < 29.0 h n:19,333	2nd 29.0–41.3 h n:19,326	3rd > 41.3 h n:19,358
Procurement interval, hours	8.1 (7.2–8.6)	10.5 (9.9–11.3)	14.9 (13.2–17.9)	22.8 (18.2–26.2)	34.6 (31.8–37.7)	52.1 (45.9–62.2)
Donor age, years	59 (51–64)	51 (37–59)	47 (33–56)	45 (26–56)	38 (23–51)	35 (23–48)
Donor BMI, kg/m^2^	24.8 (23.4–27.8)	24.3 (22.0–26.3)	24.2 (22.5–26.6)	26.4 (22.8–30.7)	26.0 (22.4–30.4)	26.0 (22.6–30.2)
Donor gender, male	113 (52.8%)	109 (50.7%)	120 (56.1%)	11,136 (57.6%)	11,548 (59.8%)	11,907 (61.5%)
Donor medical history						
Hypertension	77 (36.0%)	47 (21.9%)	46 (21.5%)	7,343 (38.1%)	5,939 (30.9%)	5,121 (26.7%)
Diabetes	21 (9.8%)	6 (2.8%)	10 (4.7%)	2,329 (12.0%)	1,821 (9.4%)	1,579 (8.2%)
Donor cause of death						
Anoxia	0 (0.0%)	4 (1.9%)	12 (5.6%)	5,668 (29.3%)	5,823 (30.1%)	6,282 (32.5%)
Cerebrovascular accident	163 (76.2%)	143 (66.5%)	137 (64.0%)	7,393 (38.2%)	6,149 (31.8%)	5,291 (27.3%)
Trauma	44 (20.6%)	62 (28.8%)	57 (26.6%)	5,885 (30.4%)	6,874 (35.6%)	7,226 (37.3%)
Other	7 (3.3%)	6 (2.8%)	8 (3.7%)	387 (2.0%)	480 (2.5%)	559 (2.9%)
Donor Risk Index^a^	1.52 (1.38–1.77)	1.42 (1.16–1.65)	1.35 (1.16–1.52)	1.33 (1.11–1.61)	1.26 (1.08–1.52)	1.22 (1.08–1.47)
More than liver and kidney donor^b^	29 (13.6%)	84 (39.1%)	145 (67.8%)	7,240 (37.4%)	11,204 (58.0%)	13,220 (68.3%)
Thoracic donor	12 (5.6%)	68 (31.6%)	124 (57.9%)	6,542 (33.8%)	10,511 (54.4%)	12,753 (65.9%)
Recipient						
Partial/split graft	11 (5.1%)	19 (8.8%)	25 (11.7%)	312 (1.6%)	525 (2.7%)	628 (3.2%)
Cold ischemia, hours	4.74 (4.22–5.70)	4.93 (4.28–5.70)	4.95 (4.38–5.68)	6.0 (4.7–7.7)	6.3 (5.0–8.0)	6.0 (4.8–7.7)
Recipient age at transplantation, years	55 (44–60)	51 (32–60)	50 (34–59)	56 (48–61)	56 (47–62)	56 (46–62)
Recipient MELD at transplantation	16 (11–23)	15 (11–21)	15 (10–20)	21 (14–30)	21 (13–30)	22 (13–33)
Liver pathology						
Acute liver failure	31 (14.5%)	24 (11.2%)	17 (7.9%)	952 (4.9%)	881 (4.6%)	978 (5.1%)
Malignancy	33 (15.4%)	36 (16.7%)	32 (15.0%)	4,885 (25.3%)	4,788 (24.8%)	4,829 (24.9%)
PSC	25 (11.7%)	39 (18.1%)	45 (21.0%)	788 (4.1%)	799 (4.1%)	852 (4.4%)
Alcoholic liver disease	44 (20.6%)	29 (13.5%)	38 (17.8%)	3,137 (16.2%)	3,227 (16.7%)	3,596 (18.6%)
Other	81 (37.9%)	87 (40.5%)	82 (38.3%)	9,571 (49.5%)	9,631 (49.8%)	9,103 (47.0%)
Year of Transplant	2011 (2007–2014)	2010 (2007–2014)	2012 (2009–2016)	2011 (2009–2014)	2013 (2011–2016)	2015 (2013–2017)
Follow-up time, years	7.15 (3.97–11.78)	6.65 (3.38–10.89)	5.34 (3.11–9.06)	4.1 (1.3–7.0)	3.0 (1.0–5.9)	1.9 (0.6–3.9)

a Formula by Feng et al (12).

b Organs donated besides liver and kidneys, categorical.

All values are stated as median (interquartile range) or exact number (percentage of all) unless otherwise indicated.

**TABLE 3:** Outcomes of liver transplantations in Finland and the US by tertiles of time between brain death and organ procurement (interval).

Variable	Finland 6/2004–12/2017			US 1/2008–8/2018		
Tertile of procurement interval	1st <9.2 h n:214	2nd 9.2–12.0 h n:215	3rd >12.0 h n:214	1st <29.0 h n:19,333	2nd 29.0–41.3 h n:19,326	3rd >41.3 h n:19,358
Graft survival						
1-year	92.1%	90.2%	92.5%	87.0%	88.2%	89.3%
3-year	84.6%	83.1%	87.8%	78.5%	80.3%	82.5%
5-year	81.4%	77.0%	84.4%	72.6%	74.7%	76.5%
10-year	74.6%	64.2%	77.0%	57.2%	61.9%	58.9%
15-year	57.2%	46.4%	66.0%	NA	NA	NA
Intrahepatic biliary stricture	7 (3.3%)	13 (6.1%)	11 (5.2%)	NA	NA	NA
Anastomotic biliary stricture	33 (15.4%)	27 (12.6%)	31 (14.5%)	NA	NA	NA
Biliary leak	4 (1.9%)	7 (3.3%)	7 (3.3%)	NA	NA	NA
Discharge time	NA	NA	NA	10 (7–18)	10 (7–18)	11 (7–19)
Early acute rejection^a^	44 (20.5%)	56 (26.2%)	52 (24.3%)	1,116 (5.8%)	1,012 (5.3%)	970 (5.1%)
Acute rejection during first year^b^	73 (34.1%)	84 (39.1%)	74 (34.6%)	1,122 (14.4%)	1,137 (14.6%)	1,159 (14.8%)
MEAF^c^	3.3 (2.1–4.6)	3.3 (2.0–4.5)	2.9 (1.6–4.1)	NA	NA	NA
Post-operative dialysis^d^	25 (14.1%)	30 (16.7%)	30 (16.5%)	NA	NA	NA
Post-operative kidney injury^e^	124 (70.5%)	122 (67.8%)	121 (66.5%)	NA	NA	NA
Difference in creatinine^f^	57 (18–131)	40 (14–121)	45 (15–113)	NA	NA	NA

a In Finnish cohort acute rejection before 30 days and in the US cohort before discharge.

b For US in sub-cohort of transplantations performed 2013 onwards (middle-tertile of 33–46 h of procurement interval).

c Model for Early Allograft Function (13), median (interquartile range).

d AKI requiring dialysis within 7 post-operative days.

e Acute kidney injury defined by KDIGO guidelines, grades 1–3. 104 patients (16.2%) were dialysed preoperatively and were excluded from post-operative kidney injury and dialysis analysis.

f Difference between highest creatinine in 7 post-operative days and pretransplantation creatinine in mmol/l.

### Short-Term Clinical Outcomes

#### Biliary Strictures, Acute Rejections and Kidney Injury as Outcome in the Finnish Cohort

In the Finnish cohort, 31 patients had intrahepatic biliary strictures during follow-up. 18 of these occurred in patients with primary sclerosing cholangitis (PSC), five with acute liver failure, two with alcoholic liver disease, one with liver malignancy, and five in patients with other liver pathologies as the indication for liver transplantation. In a univariable logistic regression model with spline, procurement interval was not associated with intrahepatic strictures (*p* = 0.65 for non-linearity in univariable analysis, *p* = 0.78 for linear component, OR 1.08 95% CI 0.76–1.54). No association was found in a multivariable logistic regression model (*p* = 0.36 for non-linearity, linear OR 0.99 95% CI 0.67–1.46).

During the first year after transplantation, 231 of 643 (36%) patients had an acute rejection episode. In a univariable logistic regression model with spline, the association of procurement interval to acute rejection during first year was not significantly non-linear (*p* = 0.31) and in a linear model failed to show statistical significance (OR 1.15 95% CI 0.98–1.36). In the adjusted model the association stayed insignificant (*p* = 0.29, OR 1.11 95% CI 0.92–1.34). Early acute rejections in the first 30 post-operative days were in a linear univariable model associated with longer procurement interval (*p* = 0.024, OR 1.23 95% CI 1.03–1.47). This association was lost in a multivariable model (*p* = 0.16, OR 1.16 95% CI 0.94–1.42).

From the kidney injury analysis, 104 (16.2%) patients were excluded having been dialyzed preoperatively. 85 patients required dialysis during the first seven post-operative days after transplantation. In a univariable logistic regression model with spline, the association of procurement interval to kidney injury requiring dialysis failed to show non-linearity (*p* = 0.62) or significant linear association (OR 1.02 95% CI 0.79–1.31), which was the case for the multivariable model as well (linear model OR 1.09 95% CI 0.82–1.44). Similarly, when defined by acute kidney injury (AKI) grade 1, 2 or 3 of KDIGO guidelines, kidney injury was not associated with procurement interval (non-linearity *p* = 0.64 and *p* = 0.70, linearly OR 0.99 95% CI 0.81–1.21 and OR 1.03 95% CI 0.82–1.30 in univariable and multivariable model, respectively). Univariable logistic regression model probabilities of endpoints are represented with a spline function by procurement interval in Supplementary Figure S2, which sums the results regarding the Finnish cohort short-term logistic regression results.

#### MEAF-Score as Outcome in the Finnish Cohort

MEAF-score could not be calculated for six (0.9%) patients due to missing laboratory results and 4 patients due to death before third post-operative day. For this analysis, 65 underaged, 4 partial grafts and 68 acute liver failures were excluded for lack of validation of MEAF in these cohorts. Median MEAF in the remaining 496 complete cases was 3.19 (IQR 1.93–4.39). Longer procurement interval associated with better MEAF-scores (*p* = 0.021, Beta −0.018 95% CI −0.079 −(−0.006)) in a univariable linear model and in a multivariable model (*p* = 0.005, Beta −0.048 95% CI −0.085 −(−0.011)). A linear regression curve with confidence intervals is portrayed with a scatter plot of MEAF over procurement interval in Supplementary Figure S3.

#### Acute Rejections as Outcome in the US Cohort

**FIGURE 2:**
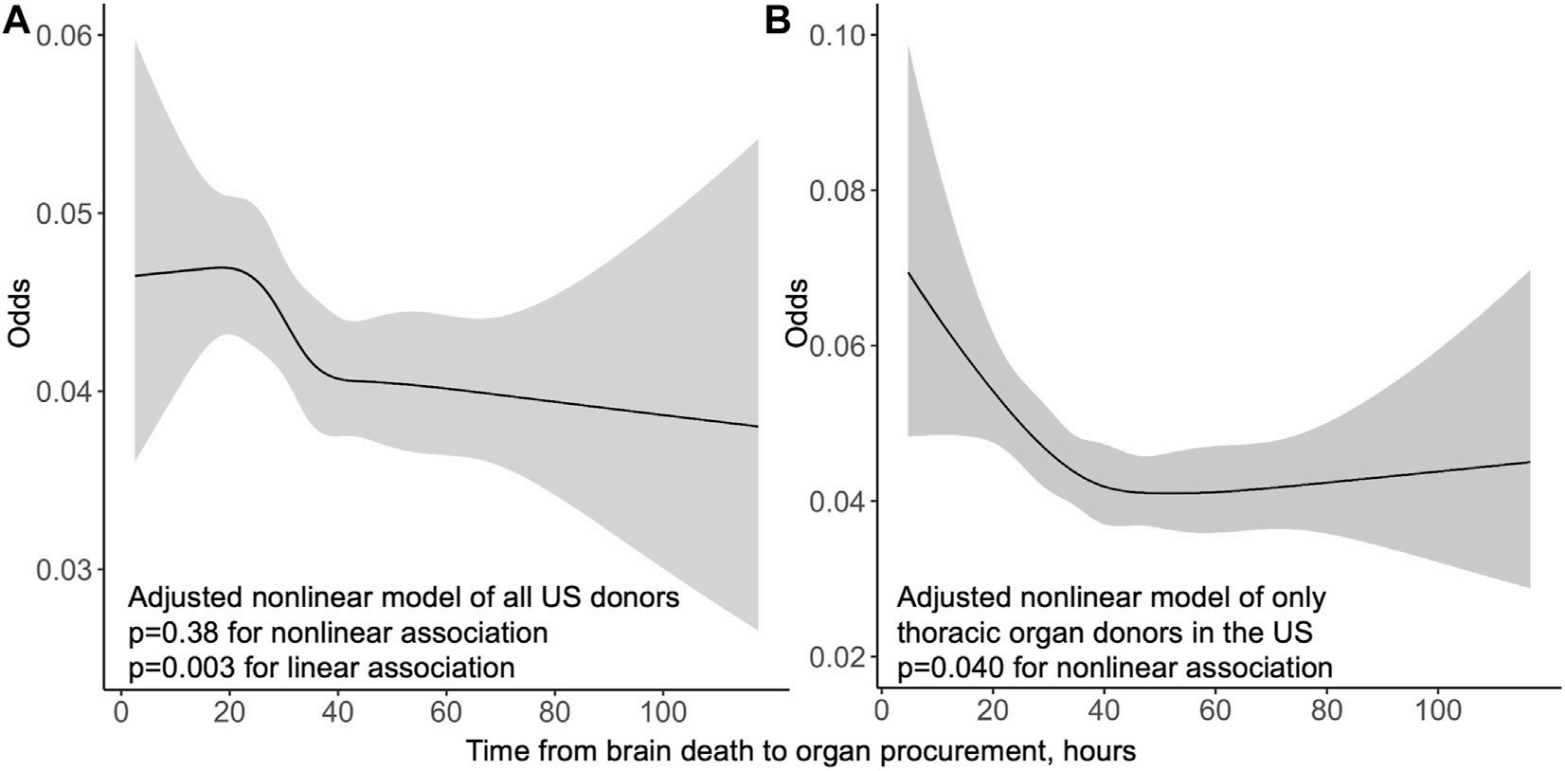
Odds of acute rejection before discharge by procurement interval from multivariable logistic regression models in the US whole cohort **(A)** and thoracic organ donors only **(B)** with 95% confidence intervals in grey.

Of 57,966 transplants 3,102 (5.4%) suffered an early acute rejection before discharge time, which was median 10 days (IQR 7–18 days). Longer procurement interval was linearly associated with lower risk for early acute rejection in univariable analysis (*p* = 0.005, OR 0.939 per 1 hour longer interval, 95% CI 0.899–0.981) and in multivariable model (*p* = 0.003, OR 0.935, per 1 hour longer interval, 95% CI 0.894–0.978) (Figure 2).

Acute rejections during the first year were analysed only from 2013 forward due to missing data. Restricting the cohort to transplantations from 2013 forward and to cases with complete 1-year follow-up of acute rejections, a total 23,430 transplantations were included for this sub-group analysis. 3,418 (14.6%) patients had an acute rejection episode before 1-year follow-up. No significant association of procurement interval with acute rejections during first year was detected (*p* = 0.36 for non-linearity, OR = 1.01 95% CI 0.97–1.06 for univariable model, OR 1.00 95% CI 0.95–1.04 for multivariable model).

### Graft Survival

#### Finnish Cohort

**FIGURE 3:**
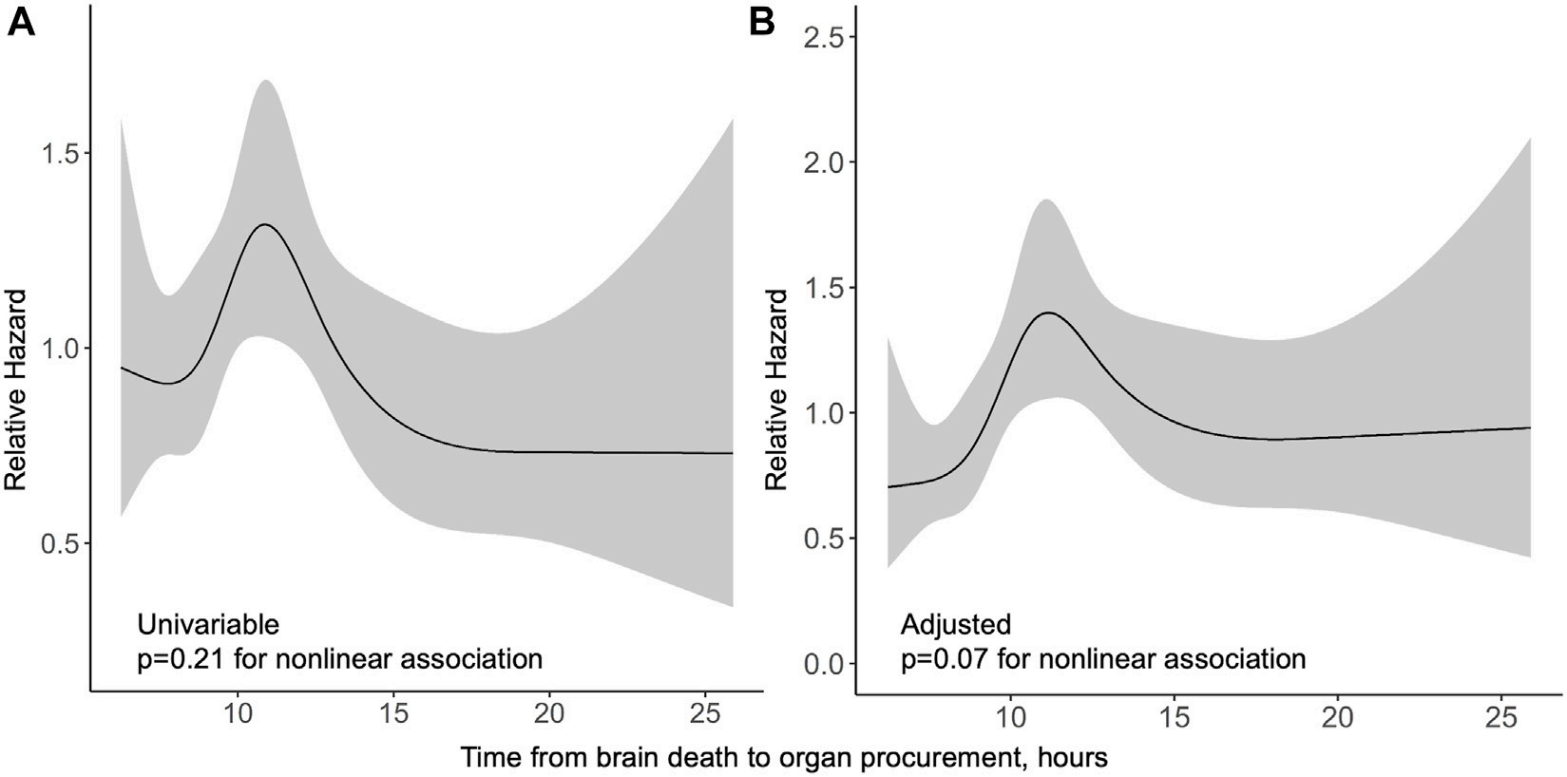
Relative hazard of graft loss or death by procurement interval from univariable **(A)** and multivariable **(B)** Cox regression models in the Finnish cohort with 95% confidence intervals in grey.

In the Finnish cohort, procurement interval was not significantly associated with graft survival. In a univariable spline model, procurement interval was not associated with graft survival non-linearly (*p* = 0.21) or linearly (*p* = 0.44, HR 0.99 95% CI 0.95–1.02). The relative hazards of both univariable and multivariable model are presented in Figure 3. Non-linear association of procurement interval with graft survival did not reach statistical significance in a multivariable model (*p* = 0.07) and no linear association was found (*p* = 0.45, HR 1.01 95% CI 0.98–1.05). Non-proportionality was tested for and held in a univariable model (*p* = 0.76) and in the multivariable model (*p* = 0.72) for procurement interval.

#### US Cohort

**FIGURE 4:**
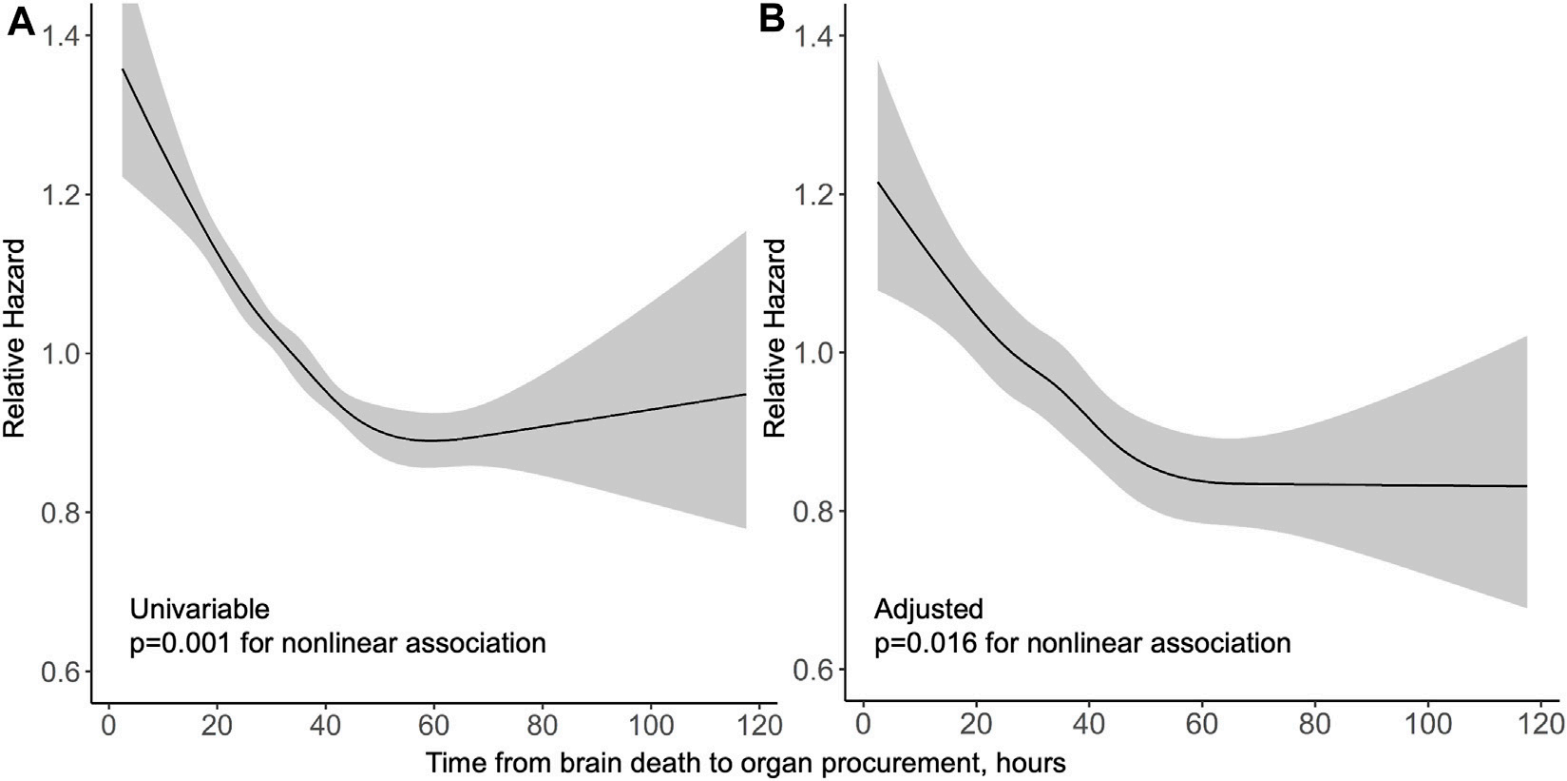
Relative hazard of graft loss or death by procurement interval from univariable **(A)** and multivariable **(B)** Cox regression models in the US cohort with 95% confidence intervals in grey.

**FIGURE 5:**
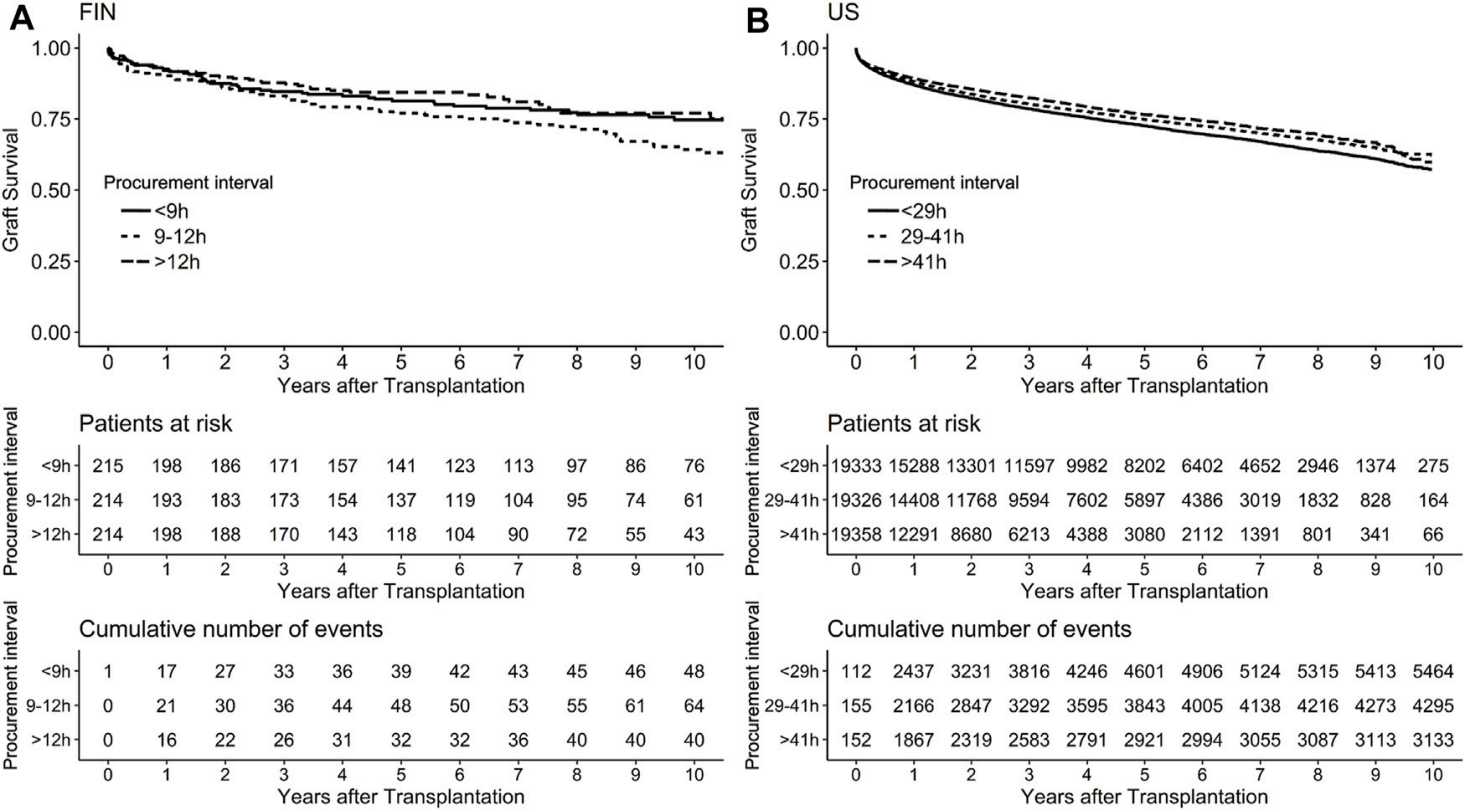
Kaplan-Meier curves with survival tables in Finnish **(A)** and the US **(B)** cohort with cases divided by procurement interval tertiles for graphical purposes with 95% confidence intervals in grey.

Median follow-up period in the US was 3 years. In a univariable model, the association of procurement interval with graft survival showed strong non-linearity (*p* < 0.001) and is presented by a cubic spline function of relative hazard in Figure 4. Longer interval associated non-linearly (*p* = 0.016) with better graft survival also in multivariable models adjusted with Donor Risk Index (DRI) and recipient factors (age, MELD and acute liver failure) (Figure 4). Proportional hazards assumption held true for procurement interval (*p* = 0.20). Kaplan-Meier curves of both cohorts are presented in Figure 5.

### Sensitivity Analyses

#### Organ Yield

All analyses were repeated with stratification to donor organ yield. Cohorts were separated by whether thoracic organs were donated or not (thoracic donor) resulting in two sensitivity analyses by organ yield. In the Finnish cohort, no new associations of short-term outcomes were detected. Linear association of longer procurement interval with decreasing MEAF-score was barely lost in thoracic donors (univariable *p* = 0.072, Beta −0.060 95% CI −0.125–0.005) and multivariable model (*p* = 0.070 Beta −0.051 95% CI −0.115–0.013), and no significant association was detected for donors who donated only abdominal organs (*p* = 0.23). Otherwise, the sensitivity analyses by organ yield concurred with results for the Finnish cohort.

In the US cohort, stratification to thoracic donors resulted in increasing variance in short and very long procurement interval associations with graft survival, leading to dissipated non-linearity (*p* = 0.22) (Supplementary Figure S4). Linear decreasing hazard remained (HR 0.910 95% CI 0.880–0.942) with longer procurement interval with stratification to thoracic donors in a multivariable model. The non-linear association of procurement interval with graft survival observed in the whole cohort persisted with non-thoracic donors (*p* = 0.008). Stratification to thoracic donors yielded a non-linear association of longer procurement interval with less acute rejections before discharge (*p* = 0.040) (Figure 2). This association of procurement interval with acute rejections disappeared entirely when the cohort was restricted to non-thoracic donors (adjusted non-linear association *p* = 0.99, linear *p* = 0.15).

#### Transplant Year

Procurement intervals grew longer during the follow-up in both cohorts (Table 2). A sensitivity analysis was conducted in the US cohort by dividing transplantations to two groups: 2008–2012 and 2013–2018 to account for this possible confounding. For both sub-cohorts of US cohort, non-linearity of association of procurement interval with graft survival was lost due to growing of confidence intervals in shorter procurement intervals (Supplementary Figure S5). Linear decreasing adjusted hazard of graft loss or death was significant in both sub-cohorts (earlier transplantations *p* = 0.002, HR 0.962 95% CI 0.939–0.985, and later *p* = 0.028, HR 0.959 95% CI 0.924–0.995). The association of procurement interval with acute rejections before discharge disappeared for both earlier and later sub-cohorts (adjusted linear association *p* = 0.051 and *p* = 0.77, respectively).

In the Finnish cohort, sensitivity analyses by transplantation year groups were 2004–2011 and 2012–2017. Longer procurement interval was associated with better MEAF-scores (*p* = 0.002, Beta −0.074 95% CI −0.120 −(−0.028)) only in the later years reflecting the change to longer procurement intervals (in earlier transplantations adjusted *p* = 0.80). When the Finnish cohort was divided to earlier and later transplantations, new associations of procurement interval with other outcomes were not detected concurring with whole cohort analyses. In Supplementary Figure S6 a spline function represents the association of procurement interval with relative hazard of graft loss or death for earlier and later transplantations in the Finnish cohort.

#### 1-year Graft and Patient Survival

When the follow-up was restricted to 1 year after transplantation, the results concerning the composite endpoint of graft and patient survival remained the same. In the Finnish cohort, the association remained insignificant (Supplementary Figure S7). In the US cohort, the association of the composite endpoint with procurement interval was non-linear (*p* = 0.0036) in the multivariable Cox model—the relative hazard diminishing until 60 h after brain death (Supplementary Figure S8). The sample size of US cohort enabled us to analyze separately solely graft- and patient survival 1 year after transplantation. For both separate endpoints—solely graft and patient survival—the association of procurement interval remained non-linear in the multivariable analysis (*p* = 0.0030 and *p* = 0.0023) (Supplementary Figures S9, S10).

## Discussion

This study shows that longer procurement interval is associated with better liver graft survival and early function. The association with graft survival was only detected in the US cohort, where procurement intervals were considerably longer compared to the Finnish cohort. The shorter procurement intervals in Finland possibly fail to grasp this beneficial association seen in the US cohort. In addition, longer procurement interval showed no negative association with short-term outcomes. In contrast, a slight but significant association of longer procurement interval with better early allograft function was detected and also 1-year graft and patient survival showed a similar decreasing hazard. These results imply, that longer interval is not detrimental to the allograft and instead, it may benefit early function and longevity of the liver graft.

These short- and long-term results provide support to the trend of increasing procurement intervals over the years, which was observed in both cohorts. The reasons to lengthening procurement intervals seem logistics-driven. Due to improved donor management, the need of urgent procurement from an unstable donor has undoubtedly decreased and thus, also contributes to longer intervals. In addition, earlier studies negating harm to other organs may also have contributed.

In human studies, no organ has benefited from a very short procurement interval. In kidney allografts, four studies have reported improved graft survival with longer interval while one smaller study found no association either way (7, 8, 11, 17, 18). In heart allografts, longer procurement interval has not benefited nor harmed graft survival (10, 19). While lung transplants showed no association of procurement interval with graft survival, they benefited from longer interval with less acute rejections and bronchiolitis-obliterans-free survival (9). In these other studies on kidneys and hearts, procurement interval has not associated with acute rejections.

Unquestionably brain death is harmful for organs. Although changes in blood coagulation, cytokine profiles, and gene transcription (20) are widely recognised, time-dependent changes in relation to brain death have rarely been reported. Danobeitia et al showed in rhesus macaques that the massive catecholamine storm dies down after 6 h from brain death (21). In a novel human study, Schwartz et al showed for the first time how different cytokines fluctuate several hours after brain death (22). In their study, procurement was performed at median time of 15 h. Cytokines Interleukin-1B and Interleukin-10 increased until 7 h after brain death and stayed level until procurement. Tumour Necrosis Factor peaked at 7 h, while Interferon-gamma in turn started increasing only after 7 h after brain death. Cytokine storm seems to continue after catecholamine storm subsides, although no explicit serial data on humans exist. These and earlier studies concerning procurement intervals have led to the two-hit theory of brain death, with a catecholamine storm followed by “storm cooling,” and recovery before the second hit of cold ischemia, for which the organ is probably more prepared for after a longer procurement interval. This study is in line with this theory presented first by Kunzendorf et al (8, 17). The mechanisms are beyond the scope of this study but could be related to the upregulation of cytokines and cytoprotective genes caused by brain death similarly to the theory behind remote ischemic preconditioning, which is also being actively investigated in the field of transplantation (23).

A concern in waiting for long periods prior to procurement has been the possible loss of unstable donors and hence valuable organs. Donor management protocols have in recent decades however made this concern practically irrelevant as few potential donors are lost due to cardiovascular collapse (6, 24–28).

This study has some limitations. Firstly, causality cannot be concluded from an observational registry analysis. Due to the retrospective nature of the study, it is also susceptible to confounding and non-random allocation, which concern all the previous studies as well. Confounding is most evident in possibly changed clinical practices over the years with simultaneous lengthening of procurement intervals, which cannot be adjusted for in expense of follow-up time. This possible confounding was negated by the sensitivity analysis conducted, although limiting the association to only linear connections. Finnish cohort sample size and narrow distribution of procurement intervals, especially concerning non-multiorgan donors, also limits our ability to adjust our model and divide to sensitivity analyses.

As only the time of declaration of brain death was available to us from the cohorts, we chose to use this time for the start of the interval, although the exact time of the brain insult is unknown. Practises in different systems and countries may also differ on the urgency of diagnosing brain death. In some cases, a suspected donor will need to be stabilised before attaining the diagnosis and this can create a delay in the start of the interval. The procurement interval presented here serves therefore as the best available marker of the physiologic interval. A selection bias is unavoidable as better quality organs were distributed to longer procurement times possibly due to allocation and testing of thoracic organs. We sought to limit this with a multivariable analysis and sensitivity analyses.

The strengths of this study are the comprehensive multivariable analyses, which account for better quality organs distributing unevenly between procurement intervals, great sample size as a whole, and two cohorts with different procurement intervals offering a wider scope to the associations. One of the strengths of this research is that the findings to the same direction were found in two different populations. Since associations or effects in nature are seldom linear, spline functions were used to account for non-linearity. The peaking hazard in Finnish cohort graft survival, although insignificant, is interesting since most organs are procured exactly at the peak. Bias in this peak cannot be ruled out. Differences in patient characteristics between the cohorts were evident and practises to declare time of brain death may differ between countries; thus cohorts were analyzed separately and meant to complement each other rather than compare the cohorts. Also, practices in diagnosing and treating acute rejections may vary greatly between centers.

This analysis is to our knowledge the first to show that liver grafts may tolerate longer procurement intervals, as longer time from brain death to procurement was associated with improved outcomes. Our findings do not support a progressive organ injury induced by the cytokine storm.

## Data Availability

The datasets presented in this article are not readily available because restrictions issued by the authorities in Finland apply to the availability of the data from transplant patients for sharing. Restrictions apply to the availability of the US data based on the current data use agreements with SRTR. Other data are available from the corresponding author upon reasonable request. Requests to access the datasets should be directed to ville.sallinen@hus.fi.

## Abbreviations

DRI, donor risk index; HR, hazard ratio; IQR, interquartile range; MEAF, Model for Early Allograft Function; OR, odds ratio; SRTR, Scientific Registry of Transplant Recipients.
