# Supplementary material for: Timing of Organ Procurement From Brain-Dead Donors Associates With Short- and Long-Term Outcomes After Liver Transplantation
Source: Transpl Int. 2022 Aug 31;35:10364. doi: 10.3389/ti.2022.10364 (PMC9472133; doi:10.3389/ti.2022.10364)
Supplement: Supplementary file 1 [file DataSheet1.docx]

**Supplementary material**

**Table of Contents**

[**Supplementary Figure 1.** 1](#_Toc105591230)

[**Supplementary Figure 2.** 1](#_Toc105591231)

[**Supplementary Figure 3.** 2](#_Toc105591232)

[**Supplementary Figure 4.** 2](#_Toc105591233)

[**Supplementary Figure 5.** 3](#_Toc105591234)

[**Supplementary Figure 6.** 3](#_Toc105591235)

[**Supplementary Figure 7.** 4](#_Toc105591236)

[**Supplementary Figure 8.** 4](#_Toc105591237)

[**Supplementary Figure 9.** 5](#_Toc105591238)

[**Supplementary Figure 10.** 6](#_Toc105591239)

**Supplementary Figure 1.** Directed acyclic graph of confounders needed to account for.


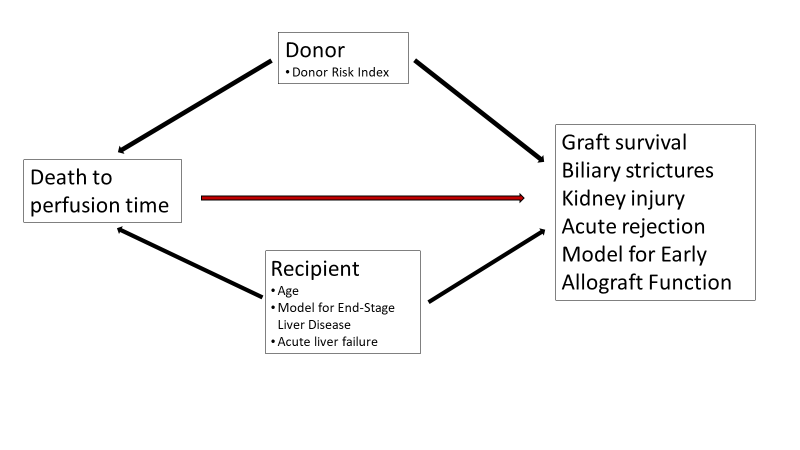


**Supplementary Figure 2.** Procurement timing associations with short-term outcomes in the Finnish cohort with cubic spline functions of probabilities of outcome.

**Supplementary Figure 3.** Linear association of procurement timing with Model for Early Allograft Function-score in the Finnish cohort (A) and with a robust model line (B).


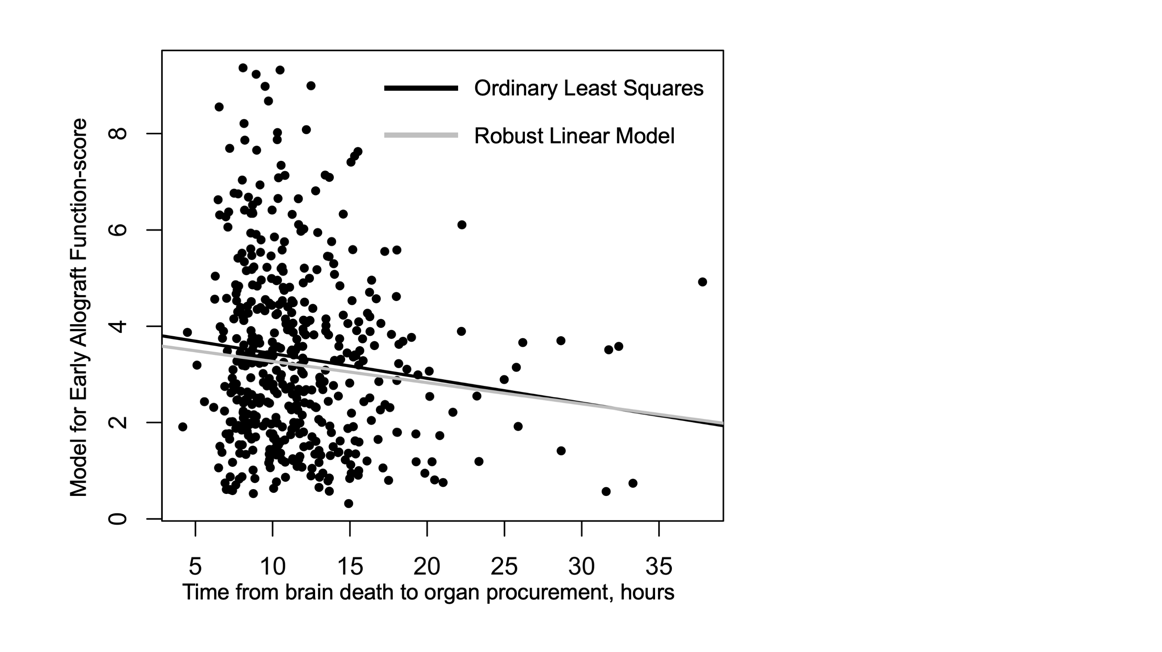


(B)

(A)

**Supplementary Figure 4.** Relative hazard of graft loss or death by procurement timing of US liver transplants when restricted to either non-thoracic donors (A) and thoracic donors (B).


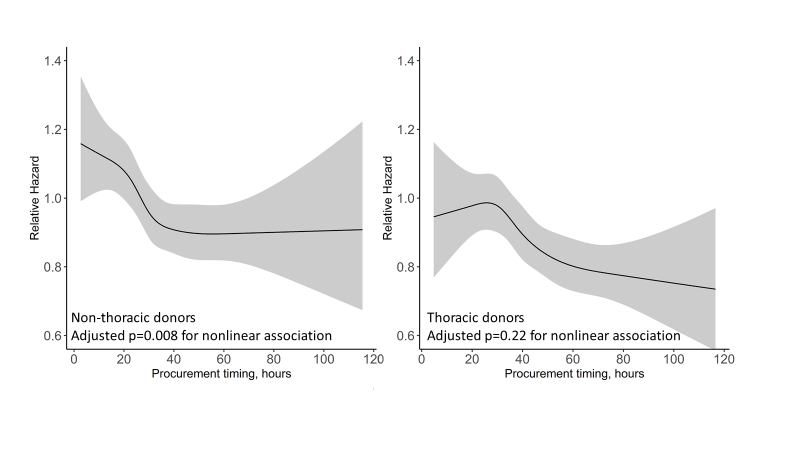


(B)

(A)

**Supplementary Figure 5.** Association of relative hazard of graft loss or death with procurement timing in US liver transplants divided to sub-cohorts of transplants performed between 2008-2012 (A) and 2013-2018 (B).


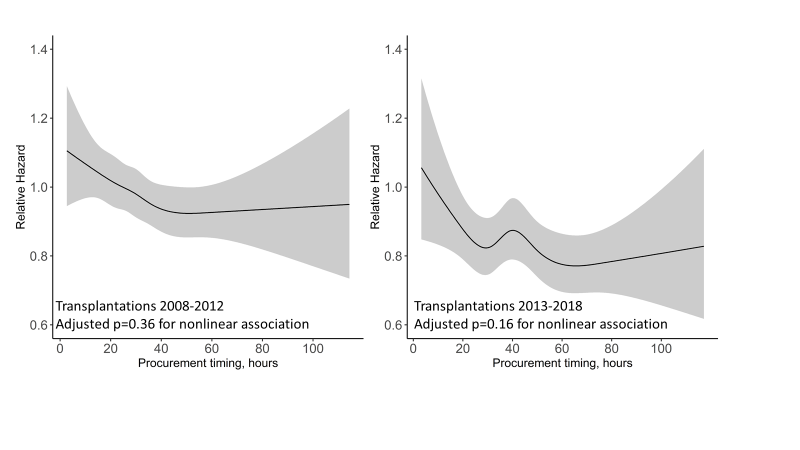


(B)

(A)

**Supplementary Figure 6.** Association of relative hazard of graft loss or death with procurement timing in Finnish liver transplants divided to sub-cohorts of transplants performed between 2004-2011 (A) and 2012-2017 (B).


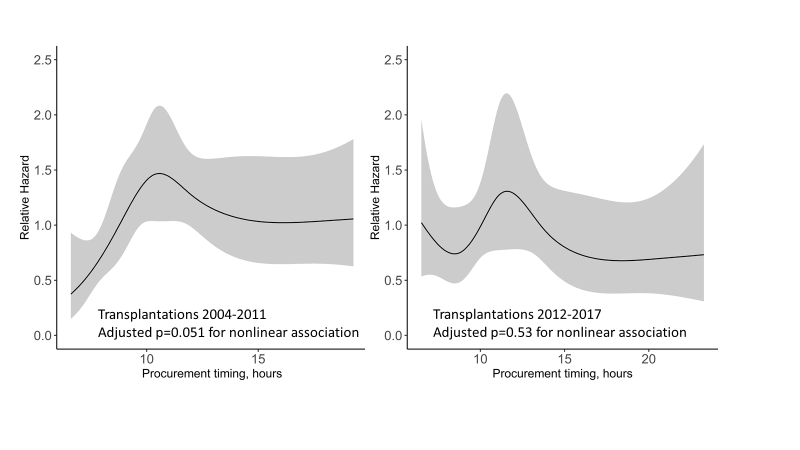


(A)

(B)

**Supplementary Figure 7.** Association of procurement timing with composite endpoint of 1-year graft survival or death after liver transplantation in Finland in a univariable (A) and a multivariable model (B).

(B)

(A)

**Supplementary Figure 8.** Association of procurement timing with composite endpoint of 1-year graft survival or death after liver transplantation in the US in a univariable (A) and a multivariable model (B).

(A)

(B)

**Supplementary Figure 9.** Association of procurement timing with 1-year graft survival after liver transplantation in the US in a univariable (A) and a multivariable model (B).

(B)

(A)

**Supplementary Figure 10.** Association of procurement timing with 1-year patient survival after liver transplantation in the US in a univariable (A) and a multivariable model (B).

(B)

(A)
